# Therapeutic effects of herbal medicines in different types of retinopathies: A systematic review

**DOI:** 10.22038/AJP.2022.62423.2977

**Published:** 2023

**Authors:** Negin Ansari-Mohseni, Adel Ghorani-Azam, Seyed Ahmad Mohajeri

**Affiliations:** 1 *Student Research Committee, Mashhad University of Medical Sciences, Mashhad, Iran*; 2 *Department of Forensic Medicine and Toxicology, School of Medicine, Urmia University of Medical Sciences, Urmia, Iran*; 3 *Pharmacetical Research Center, Pharmaceutical Technology Institute, Mashhad University of Medical Sciences, Mashhad, Iran*; † * Equal first author*

**Keywords:** Retinopathy, Retino-protective, Medicinal plant, Clinical trials

## Abstract

**Objective:**

Retinopathy is an ocular manifestation of systemic diseases such as diabetes and vascular diseases. Herbal drugs have been considered as an effective therapeutic option with minimal side effects for the treatment of retinopathy by reducing the symptoms and improving visual acuity. The purpose of this systematic review was to collect studies on the effectiveness of medicinal plants in the treatment or prevention of retinopathy.

**Materials and Methods:**

A systematic literature search was performed in PubMed, Scopus, Google Scholar, and other databases in April 2021 using “herbal products” and “Retinopathy” with all their equivalent and similar terms. For this purpose, human clinical trials with the English language were included and articles with subject irrelevancy were excluded from further evaluation.

**Results:**

Overall, 30 articles with 2324 patients were studied for possible effects of herbal therapy on retinopathy. From 30 included articles, different herbal products had been evaluated. Out of 30 selected articles, 11 articles were for the treatment of age-related macular degeneration (AMD), 14 articles covered patients with diabetic retinopathy, and the other five studies were for other retinal disorders. The outcomes in majority of the studies include changes in visual acuity (VA), fundus performance, best-corrected visual acuity (BCVA), central macular thickness (CMT), focal electroretinogram (fERG), supplements and adjuvant medications appeared to be more beneficial in patients with AMD and diabetic maculopathy.

**Conclusion:**

Herbal therapy can be considered as a potential candidate in the adjuvant and complementary therapies of retinopathy. However, further studies are required to verify such efficiency.

## Introduction

Retinopathy is an ocular manifestation of systemic diseases such as diabetes mellitus and vascular diseases that damages the retina (Nentwich and Ulbig, 2015), and can lead to partial or complete loss of vision. Diabetic retinopathy (DR) is a condition in which diabetes is responsible for the pathogenesis of the disease and is typically divided into non-proliferative (NPDR) and proliferative diabetic retinopathy (PDR). In the proliferative type, a high or fast rate of angiogenesis causes neovascularization, while in the non-proliferative type, abnormal blood flow to the retina occurs due to direct damage or compromise of the blood vessels themselves (Tarr et al., 2013). The main symptom of retinopathy is visual loss, which by itself is not localized and may be due to a defect at any location of the visual pathway (Landau and Kurz-Levin, 2011). Receptor degeneration, DR, and retinal ganglion cell disease are the main categories of retinal-related diseases. 

Retinopathy is diagnosed by an eye examination and looking through the pupil with a light, and stereoscopic fundus photography is the gold standard for the diagnosis of retinopathy (Salz and Witkin, 2015). Different diseases can cause retinopathy, leading to various conditions such as DR, retinopathy of prematurity (ROP), age-related macular degeneration (AMD), hypertensive retinopathy, etc. DR and ROP are the two most common causes of retinopathy (Penn et al., 2008). According to reports, DR affects about 5 million people and ROP affects about 50,000 premature infants each year worldwide (Akkawi et al., 2019). Hypertensive retinopathy is the next most common cause affecting 3 to 14% of all non-diabetic adults, and AMD is the most common cause of severe visual loss and blindness in people over 50 years of age (Katsi et al., 2012). Treatment is based on the cause of the retinopathy, and laser photocoagulation therapy is the standard treatment for many types of retinopathies. Using anti-vascular endothelial growth factor (VEGF) drugs has shown a significant reduction in the extent of vessel outgrowth (Park and Roh, 2016). Evidence shows that the use of anti-VEGF drugs, such as bevacizumab or pegaptanib, seems to improve the efficacy when combined with laser therapy to treat retinopathies. However, the long-term systemic effects are unclear (Sankar et al., 2018; Zhao and Singh, 2018). 

The World Health Organization (WHO) estimates that 80% of the populations of Asia and Africa use the natural medicinal drug for various therapeutic purposes. At least 7,000 pharmacologically active compounds have been derived from medicinal plants and flowers (Sofowora et al., 2013). Although herbal therapy is not without side effects, one of the major advantages of herbal drugs is the lower side effects compared to chemically synthetic medications (Balarastaghi et al., 2022; Karimi et al., 2015). To prevent the secondary complications, numerous attempts have been made to develop new therapeutic modalities to minimize unwanted drug side effects (Ghorani-Azam et al., 2018). So far, various medicinal plants and herbal products have been introduced to treat DR, most of which reduce the symptoms, improve visual acuity and visual field, and enhance the observations under ophthalmoscopy (Kumar et al., 2021). Phyto-medical studies demonstrated that Ruscus extract tablet, Sanqi Tongshu capsule, Danshen dripping pill, and injection of tetramethylpyrazine, Xueshuantong, Xuesaitong, and Puerarin could improve ischemia in the early DR, and reduce the number of microaneurysms (Zhang et al., 2018). When it is combined with laser photocoagulation therapy, ginkgo dipyridolum injection demonstrated an add-on effect, reduced the related adverse effect, and improved the patients' satisfaction (Vasant More et al., 2017). Moreover, Ginkgo (*Ginkgo biloba*), and Bilberry (*Vaccinium myrtillus*), through their antioxidant activity, contribute to the inhibition of platelet-activating factor and enhanced blood flow by decreasing blood viscosity and increasing erythrocyte deformation, which affects AMD (Chu et al., 2011; Evans, 2000; Osada et al., 2017). The purpose of this review was to summarize the clinical evidence on the role of herbal medicine in the treatment, improving the effectiveness, or reducing the adverse effects of available therapies.

## Materials and Methods

**Search strategy and selection criteria**

PubMed, Scopus, Embase, Web of Science, Medline, Ovid, Science Direct, and Google Scholar were searched for the defined keywords. The key terms for this systematic review were “Retinopathy” and “Eye disorder” and “Retinoprotective” and “Medicinal plant” including all their equivalents and similar terms. All included documents were double-checked, and to minimize the possibility of data loss, manual reference list screening of the included articles was also performed. Then, the search was limited to human studies and clinical trials. Articles irrelevant to the main purpose of this study were excluded from further evaluation. Besides, duplicated articles (articles that appeared in several databases) were excluded. Therefore, according to the abovementioned, exclusion criteria in this systematic review were as follows: I. Article with language other than English. II. All types of articles except human clinical trials. III. Articles with subject irrelevancy. IV. Duplicated documents.

Procedures including literature search and data collection were performed in April 2021 according to the PRISMA checklist 2009 by two authors independently. In the case of disagreement between the two authors in each step, the third author performed procedures to resolve the issue.

**Data collection**

All necessary data were extracted from the included articles. The data include the type of study, total numbers of participated patients, age of participants, sex ratio, and the type of retinopathy. Also, other parameters such as the name of the plant or herbal products, type of formulation, route of administration, doses of medication, the duration of therapy and the main findings were extracted. 

**Measured variables, and quality assessment of the included literature**

The primary outcomes in the majority of the studies include changes of visual acuity (VA), fundus performance, best-corrected visual acuity (BCVA), central macular thickness (CMT), focal electroretinogram (fERG), DR incidence rates, retinal thicknesses in the macular region, retinal sensitivity, hemorrhage area of the fundus, and clinical finding in which response to the therapy was defined according to changes in each criterion. According to the type of eye disorder, the outcomes in each study differ. For example, BCVA, CMT, and fERG are important in diabetic maculopathy and AMD. Also, based on the evidence in effective and positive correlation studies, the concentration of zeaxanthin and lutein in plasma, as an important factor in reducing pigment thinning and preventing the progression of the early stage of AMD, was considered as one of the variables measured in AMD patients (Chan et al., 2019; Cheng et al., 2005). To evaluate the quality of articles from all aspects such as randomization and reliability of data, two different scales including Newcastle-Ottawa, and Oxford quality scoring systems (also known as Jadad score) were used for quality assessment of the included randomized controlled trials (RCT). In the Newcastle-Ottawa scoring system, there are three different parts including “selection”, “comparability”, and “outcome” with overall 8 questions, wherein a star is given for each item. However, a maximum of two stars can be assumed for comparability, and a fully standard paper with an appropriate design can obtain a maximum of 9 stars on this scale, in which a score ≥6 has a high quality. Oxford quality assessment scoring systems contain 5 questions indicating the randomization, blinding and follow-up of studies wherein a study with a score ≥3 of 5 has a high quality. The questions of Newcastle-Ottawa and Jadad quality assessment scales are provided as supplementary data.

## Results

**Literature search and study selection**

**Figure 1:**
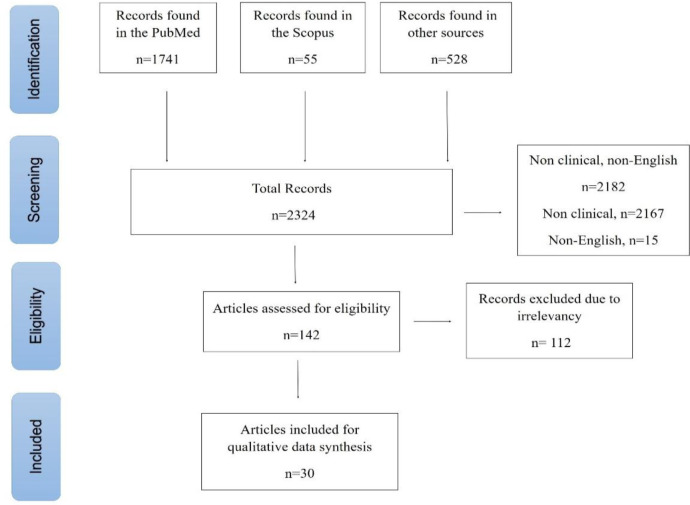
Overview of article selection process

Of 2324 articles collected in the database search, 1741 articles were from PubMed, 55 articles were from Scopus and 528 papers were from other sources (Google Scholar, Embase, Web of Science, Medline, Ovid, and Science Direct). After excluding review articles and other irrelevant documents, 30 documents were selected for further processing. Step by step process of article selection is demonstrated in Figure 1. In 14 articles, the effects of herbal medicine have been evaluated on DR, and in 11 articles, the effects of herbal therapy have been investigated on AMD, and other types of retinopathies were evaluated in 5 articles. Also, one study did not identify the type of retinopathy being studied. 

**General characteristics of the included articles**

Among the included articles, 2385 patients had been studied for possible effects of herbal therapy on retinopathy. Of the included patients, 1049 were male, 1153 were female, and the sex ratio of 183 patients had not been reported. The age range of patients was 18-89 among the studies. The minimum and maximum duration of therapy were 3 and 144 weeks, respectively. The most old and recent articles had been published in 2000 and 2020, respectively. The quality assessment of included literature is also demonstrated in Table 1.

**Study results**

Among the 30 selected studies, 24 types of herbal products were used in clinical trials to treat different types of retinal disorders. Summary of information and data of studies including disease type, herbal product, dosing, duration of treatment, type of formulation, study design, numerical results, and the first author’s name were shown in Table 2. Majority of studies were randomized, double-blind, placebo-controlled clinical study. However, in some of the studies, the control group did not receive any treatment or used other approved drugs for the treatment of retinopathy. In some cases, crossover studies were reported, and some were compared only with their baseline. The average of numerical changes of the main variables of each study or variables with significant changes was reported.

**Table 1:** Quality assessment of articles according to Newcastle-Ottawa and Oxford quality scoring systems (Jadad)

**No**	**Authors-year**	**Newcastle-Ottawa score**	**Jadad score**	**No**	**Authors-year**	**Newcastle-Ottawa score**	**Jadad score**
1	Pan et al., 2020	8	4	16	Chous et al., 2015	9	5
2	Luo et al., 2019	8	5	17	Steigerwalt et al., 2012	5	2
3	Jin et al., 2018	7	5	18	Luo et al., 2009	8	4
4	Marangoni et al., 2013	4	1	19	Steigerwalt et al., 2009	8	5
5	Piccardi et al., 2012	6	3	20	Spadea et al., 2001	5	4
6	Bucheli, P-2011	8	5	21	Rath et al., 2019	8	5
7	Falsini et al., 2010	9	5	22	Zhao et al., 2020	9	5
8	Ahmadi et al., 2009	8	4	23	Moon et al., 2019	7	5
9	Benzie et al., 2006	7	5	24	Luo et al., 2015	9	5
10	Cheng et al., 2005	8	2	25	Lian et al.,2015	9	5
11	Breithaupt et al., 2004	5	2	26	Domanico et al., 2015	8	5
12	Forte et al., 2011	8	3	27	Chan et al., 2019	8	5
13	Mazzolani et al., 2018	4	1	28	Zhu et al., 2002	4	4
14	Sepahi et al., 2018	8	5	29	Isobe et al., 2003	4	2
15	Zhao et al., 2016	8	5	30	Piccardi et al., 2019	8	5

The maximum scores for Newcastle-Ottawa and Oxford quality scales are 9 and 5, respectively

**Table 2:** Information of the clinical trials about herbal products applied for different types of retinopathies

**NO**	**Diseases**	**Patient number, (Male/ Female), age**	**Plant product**	**Variables**	**Dosage**	**Formulation**	**Therapy duration** **(weeks)**			**Study design**	**Result**																						**First author and years**	
1	AMD	38 (21/17), ≥50	Fufang xueshuantong (FXST) *(Panax notoginseng* , *Salvia miltiorrhiza,* *Astragali radix* and *Radix scrophulariae)*	thickness of CNV-PED complex, BCVA.	4500 mg/day	Oral capsule	12			Randomized controlled, pilot study /control with placebo	**Treatment gp**: Ranibizumab + FXST				**Control gp:** Ranibizumab + Placebo										p value								Pan et al., 2020	
											Thickness of CNV-PED complex changes														p<0.05									
											-72±11 µm				46±9 µm																			
											BCVA change mean														p<0.05									
											14.7±2.8				5.8±3.1																			
2	AMD	75 (43/32), 46-79	HBOD (*Astragalus propinquus*, *Angelica sinensis*, *Panax notoginseng* and…)	BCVA, CMT, Fundus hemorrhage area, fundus fluorescein leakage area, height of the lesion	200 ml per day	Decoction, Oral solution	24			Randomized controlled	**Treatment gp:** HBOD + Ranibizumab				**Control gp: **Ranibizumab										p value								Luo et al., 2019	
											Comparison of BCVA (EDTRS letter scores) changes																							
											+8				-1										p<0.01									
											Fundus hemorrhage area (papillary area)																							
											-0.46				-0.44										p<0.01									
3	AMD	144, 45-80	ZQMT (*Rheum officinal, Panax notoginseng, Salvia miltiorrhiza)*	CVA, change in thickness at the fovea, the area of lesion, fluid, area of hemorrhage, blood flow in the retina	15 tablet per day	Oral tablet	24			Randomized controlled, pilot study /control with placebo	**Treatment gp:** Ranibizumab + ZQMT				**Control gp:** Ranibizumab + Placebo										p value								Jin et al., 2018	
											Average Ranibizumab injections needed in 24 weeks																							
											0.22				0.82										p<0.0001									
											Average drug cost in both groups (US$)																							
											500				1000										<0.05									
											Relative changes in area of hemorrhage																							
											-32				-11										˂0.001									
4	AMD	33 (15/18), 51-85	Saffron (*Crocus sativus*)	fERG amplitude and macular sensitivity	20 mg/day	Oral capsule		12	Clinical trial		Treatment														p value (compared with baseline)								Marangoni et al., 2013	
											fERG modulation function (fERG amplitude log µv) changes after 12 weeks																							
											CFH (rs1061170) polymorphism																							
											Wild type	Heterozygous					Homozygous								Not shown a significant difference between genotype groups.									
											+1.2	+1.1					+1																	
											compared to baseline values (p<0.01)																							
											ARMS2 (rs10490924) polymorphism																							
											+1.1	+1.1					+1								Not shown a significant difference between genotype groups									
											compared to baseline values (p<0.01)																							
5	AMD	29 (16/13), 55-89	Saffron (*Crocus sativus*)	(fERG)-derived macular (18°) flicker sensitivity estimate, CVA	20 mg/day	oral capsule		15	Longitudinal, interventional open-label study		**Treatment** saffron														p value (compared with baseline)								Piccardi et al., 2012	
											Mean fERG sensitivity changes																							
											+0.3 log units														p<0.01									
											mean visual acuity changes																							
											0.75 to 0.9														p<0.01									
6	AMD	150 (58/92), 65-70	* Lycium barbarum L.*	Soft drusen count, plasma zeaxanthin level and antioxidant activity levels.	13.7 g/day	Oral powder lacto-Wolfberry		12	Double-masked, randomized, placebo-controlled		**Treatment gp:** Lacto-Wolfberry				**Control gp:** Placebo										p value								Bucheli et al., 2011	
											Macula pigmentation changes%																							
											**0**	**1**		**More**				**0**				**1**			**More**				<0.01								
											-3	-3		0				-9.2				-1.1			10.3												
											Soft macular drusen count changes%																							
											**0**	**1**		**More**				**0**				**1**			**More**				p=0.02								
											-0.9	-0.9		0				-6.1				-5.7			11.8												
7	AMD	30 (12/18), 54-84	Saffron (*Crocus sativus*)	fERG functions, CVA	20 mg per day	Tablet		12	Randomized controlled trial with placebo		**Treatment gp:** saffron				**Control gp:** Placebo										p value								Falsini et al., 2010	
											Mean change fERG function																							
											0.25 log µV				-0.003 log µV										˂0.01									
											Visual acuity change																							
											+0.1				+0.02										<0.01									
8	AMD	280 (157/ 123), 60-78	HESA-A **(**1-*Carum carvi* (Persian cumin), 2-*Penaeus latisculatus* (king prawn), and 3- *Apium graveolens (*celery*))*	BCVA	75 mg/Kg BID	Tablet		4	Randomized double blind clinical trial with placebo		**Treatment gp : ** HESA-A				**Control gp:** Placebo										p value								Ahmadi et al., 2009	
											Visual acuity after 1 month (LogMar) (Mean±SD)																							
											1.03±0.40				1.72±0.66										˂0.05									
9	AMD	12, 21-30	*Lycium barbarum L*	Concentration of zeaxanthin	15 mg/day	Oral powder sachet. ;;		5	Double-blinded, controlled, human intervention trial of multiple cross-over design		Formulation																						Benzi et al., 2006	
											A				B										C									
											Zeaxanthin bioavailability) Mean area under the curve (nmol * h/l																							
											9.73				3.24										1.09									
											A > B = C																							
10	AMD	27, 18-48	*Lycium barbarum L*	Fasting plasma zeaxanthin concentration changes	15 g/day in 28 days	Tablet		24	Single-blinded, placebo-controlled		**Treatment gp**				**Control gp**										p Value								Cheng et al.,2005	
											Plasma zeaxanthin changes (µmol/l)																							
											0.058 p<0.01				0.05 p>0.05										p<0.01									
11	AMD	12 (6/6), 26.8	*Lycium barbarum L*	Concentration of zeaxanthinstereoisomers	150 mg/day	Powder mix in whole-milk yoghurt		3	Randomized, single-blind cross-over study		**Group 1**				**Group 2**										p Value								Breithaupt et al., 2004	
											3R,3՜ R-zeaxanthin (µmol/l) Day1 changes														p<0.05									
											3R,3՜R-zeaxanthin dipalmitate				Non-esterified 3R,3՜R-zeaxanthin																			
											0.061				0.02																			
											3R, 3՜ R-zeaxanthin (µmol/l) Day 2 changes														p<0.05									
											Non-esterified 3R, 3՜R-zeaxanthin				3R, 3՜ R-zeaxanthin dipalmitate																			
											0.02				0.07																			
12	Diabetic cystoid macular edema without macular thickening	40 (22/18), 62.9	*Centella asiatica, Melilotus*	BCVA, mean CRT, stability of fixation at microperimetry, retinal sensitivity	*desmin* (300 mg/day), *troxerutin *(300 mg/day) with *C. asiatica *(30 mg/day) and *Melilotus* (160 mg/day)	Not reported		56	Randomized controlled trial		**Treatment gp** C. asiatica, Melilotus				**Control gp**										p value								Forte et al., 2011	
											Mean BCVA changes (ETDRS)																							
											–0.12				–0.66										p>0.05									
											CRT changes																							
											6.2				3.83										p>0.05									
											Microperimetry retinal sensitivity (dB) changes																							
											–0.27				–0.92										p<0.001									
13	Chronic DME	11 (7/4), 50-78	Meriva® **(***curcumin)*	BCVA and average macular thickness, macular edema (CSME) in fundus	500 mg BID	Oral tablet		12	Open-label study, clinical trial.		**Treatment** Meriva®														p value (Compared with baseline)								Mazzolani et al., 2018	
											mean BCVA																							
											0.4±0.2 log MAR														p=0.0072									
											Average macular thickness change (µm)																							
											–47.6														p=0.0090									
14	DME	60 (29/31), 41-82	*Crocin (*active ingredient in Saffron (*Crocus sativus*)*)*	BCVA, CMT	5 mg or 15 mg /day	Oral tablet		12	Double-masked, placebo controlled, phase 2 randomized clinical trial		**Crocin ** **5 mg**				**Crocin** ** 15 mg**						**Placebo**				p Value With crocin15 vs. Placebo								Sepahi et al., 2018	
											Mean CMT change																							
											0.06				0.01					0.51					0.005									
											Mean p value of BCVA (Log MAR) changes																							
											0.06				0.001					0.42					0.012									
15	DR	140 (70/70), 61.5	LDP (1-*Rehmanniae radix praeparata*, 2-*Corni fructus*, 3-*Dioscoreae rhizome* and…),GLT*(Ginkgo biobla)*	CIMT, DR degree by retina camera	(LDP: 8, GLT:2) TID	Tablet		144	Randomized, double-blind and placebo-controlled clinical trial		**Treatment gp:** LDP, GLT				**Control gp:** Placebo										p value								Zhao et al., 2016	
											DR Prevalence changes % after 144 weeks																							
											8.4%				4.6%										p<0.05									
16	DR	70 (26/44), 43-69	DiVFuSS (*Curcuma longa*, *Camellia sinensis* and Pycnogenol and…)	Change in DR severity and DPNSSs, Foveal and RNFL, tumor necrosis factor α (TNFα)	2 Capsule/day	Capsule		24	Randomized acebo-controlled trial, double blinded/		**Treatment gp: **DiVFuSS				**Control gp:** Placebo										p Value								Chous et al., 2015	
											MPOD changes																							
											0.1				-0.01										0.008 to <0.0001									
17	DR	77 (43/34), 54.6	Meriva® (*curcumin*)	Peripheral pressure or flow, VAR, Oedema	2 Tab (500 mg)/day	Oral tablet		4	Controlled clinical Trial		**Treatment gp** Meriva®				**Control gp**										p Value								Steigerwalt et al., 2012	
											Microcirculatory measurements (edema, VAR) changes																							
											0.62 (p<0.05) With baseline				0.1										p<0.05									
											Retinal edema changes																							
											1.4				0.01										p<0.05									
18	DR	360 (146/ 214), 18-70	Qiming granule*(Radix astragali, Radix puerariae, Radix rehmanniae,* and ...)	Changes in the retinal blood circulation time	4.5 gr TID	Capsule		12	Multi-center, randomized parallel controlled clinical trial,		**Treatment gp:** Qiming Granule				**Control gp:** CD										p value								Luo et al., 2009	
											AVCT change																							
											3.815				2.245										p<0.01									
19	DR	46 (29/17), 46-58	Pycnogenol® (*Pinus pinaster*)	Retinal edema score, retinal thickness	50 mg TID	Tablet		8	Double-blind, placebo-controlled		**Treatment gp:** Pycnogenol				**Control gp:** Placebo										p value								Steigerwalt et al., 2009	
											Visual acuity (Snellen chart) changes																							
											Mild edema	Moderate edema:				Mild edema:								Moderate edema:				p<0.05				
											+3	+3				0								0								
											Retinal edema score (1-6) changes (scale 1–6)																					
											-0.7	-0.1			-0.8					+0.1					p<0.05				
20	DR and other vascular retinal disorder	40 (16/24), 56	Pycnogenol® (*Pinus pinaster*)	VA, VF,Pattern and endothelial permeability, Functional of eye	50 mg TID	Tablet		8	Clinical trial, double blind, randomized placebo-control		**Treatment gp:** Pycnogenol®				**Control gp**: Placebo										p Value								Spadea et al., 2001	
											Mean change of Visual acuity																							
											Right eye	Left eye		Right eye					Left eye						p<0.05 for right, p<0.01 for left eye							
											0.43	0.57		0.3					0.63													
											Vascular pattern and endothelial permeability changes (Fluorangiography)																					
											Right eye		Left eye			Right eye							Left eye			Treatment with basal p<0.01						
											-0.4		-0.26			-0.2							-0.3									
21	NAION	22 (13/9), ≥18	RPh201 (extract of gum *Pistacia lentiscus*)	BCVA	20 mg S.C twice week	S.C Injection		24	Phase 2a, single-site, prospective, randomized, placebo-controlled, double-masked trial		**Treatment gp :** RPh201				**Control gp:** Placebo											p value							Rath et al., 2019	
											BCVA improvement %																							
											36.4%				12.5%										p<0.05									
22	NPDR	80 (50/30), 57-61	*Abelmoschus manihot* (Jiahua® tablet)	NPDR severity levels by fundus camera, ETDRS vision score , macular thicknesses, VEGF levels	1.8 gr TID	Tablet		24	Randomized controlled clinical trial		**Treatment gp:** Base treatment + *Abelmoschus manihot*				**Control gp:** Base treatment										p value								Zhao et al., 2020	
											ETDRS vision score change																							
											+5				-2										0.0002									
											progression rate of NPDR severity																							
											4.2%				18.7%								0.007											
23	NPDR	124 (67/57), 40-78	Proanthocyanidins of Vitis* vinifera* (Grape seed)	BCVA, IOP, change in the grade of diabetic retinopathy on FA imaging, mean thickness of CSMT and TMV, improvement in HEs	150 mg/day GSPE	Oral tablet		48	Randomized, multicenter, double-blind trial, with placebo		Treatment gp				Control gp:								p value										Moon et al., 2019	
											GSPE				CD								Placebo													
											Comparison of treatment success rate, which was defined as a decrease in hard exudates severity of more than 2 grades at 1 year, among											
											Success, n (%) changes, intension-to-treat											
											43.9				14.29								8										0.0007			
											Success, n (%) changes, per protocol											
											46.88				14.29								10.53										o. oo22			
24	NPDR	57 (21/36), 43-67	CDDP ***Salvia. miltiorrhizae, Panax notoginseng* and...)**	BCVA, fundus hemorrhage area, MN of VF, microaneurysm number, fundus fluorescein leakage area, capillary nonperfusion area	15 pill TID One CDDP pill contains 27 mg of herbal medicine**.**	Pill		12	Randomized controlled double-dummy,double-blind Study		**Treatment gp** CDDP				**Control gp** CD 500 mg								p value										Luo et al., 2015	
											Hemorrhage area (PD) change											
											0.16				0.14								p<0.05											
											BCVA (Log MAR) improve											
											0.11				0.15								p<0.05											
25	NPDR	223 (93/130), 59.3	CDDP ***Salvia. miltiorrhizae, Panax notoginseng* and...)**	Blood flow in the retina by change in FFA and Fundoscopic examination , retinopathy severity, change in visual acuity and IOP examinations	Low-dose 10 pills/day Mild-dose 20 pills/day High-dose 30pills/day	15 pill TID		24	Randomized, double-blind, placebo-controlled multicenter clinical trial		**Treatment gp: **CDDP				**Contol gp:** Placebo								p value										Lian et al., 2015	
											High-dose				Mild-dose								Low-dose																
											FFA changes after 24 weeks (Total retinal circulation time (s))											
											-1.39				-1.71								-1.10				0.22						p˂0.001					
											Fundoscopic examination data (Capillary hemangioma) Changes at 24 weeks											
											-5.61				-4.89								-1.55				3.40					p˂0.001						
26	NPDR	68 (33/35), 60.29	Diaberet®* (Pinus pinaster* and mineral)	CMT, level of ROS	1 Tab/day	Oral tablet		24	Randomized controlled trial		**Treatment gp** Diaberet®				**Control gp**								p value				Domanico et al., 2015				
											CMT changes (µm)											
											-50.33				7.86								p<0.001											
											Free oxygen radical test levels changes (Friedman test)											
											-777.11				30.57								p<0.001											
27	Retinitis pigmentosa	42 (12/30), 26-69	*Lycium barbarum L*	Sensitivity changes central VF, macular thicknesses	2 Pack per day	Pack granule (each 5 gr pack of LB granules)		48	Double-masked and placebo-controlled clinical study		Treatment gp				Control gp								p value										Chan et al., 2019	
											Visual Acuity (ETDRS)											
											HCVA: Range: −0.10 to 1.68 LCVA: Range: 0.06 to 0.86				HCVA: Range: −0.12 to 1.04 LCVA: Range: 0.12 to 1.18								p=0.001											
											Macular thickness changes (µm)											
											2.62				7.25								p=0.008											
28	RVO	42 (24/18), 32-78	Huo Xue Ming Mu (1-*radix bupleuri* 2-*fructus gardenia* 3-*cortex moutan radices and...)*	CVA, Retinal edema, retinal hemorrhage and blood flow in the retina	Dose was not reported (BID)	Decoction of herbals powder mixture		12	Randomized, control trial		Treatment gp: Huo Xue Ming Mu				Control gp: CDDP								p value										Zhu et al., 2002	
											Comparison of retinal hemorrhage absorption rate											
											90.3				72.2								p˂0.05											
29	Central retinal artery occlusion	12 (6/6), 26	Hachimi-jio-gan (1-*Rehmannia* Root 2-*Cornus* Fruits 3-*Dioscorea Rhizome* and…)	Changes in blood flow in central retinal artery occlusion by the latest ultrasonic diagnosis	23 mg BID	Pill		4	Placebo-controlled Trial cross-over		**Treatment gp: **HJG				**Control gp: **Placebo								p value										Isobe et al., 2003	
											Mean changes of Central retinal artery blood flow velocities											
											+0.21				-0.1								p<0.05											
30	Stargardt disease	31 (14/17)	Saffron (*Crocus sativus*)	fERG testing , BCVA	20 mg	Oral tablet		48	Randomized, double-blind, placebo-controlled study		**Treatment gp: **Saffron				**Control gp:** Placebo								p value										Piccardi et al., 2019	
											fERG amplitude change(in µV)											
											Non-changed				−0.18 log µV, p<0.05								p<0.05											

* CRT: central retinal thickness, DME: diabetic macular edema, AVCT: arterio-venous circulation time, AMD: age related macular degeneration, BCVA: best corrected visual acuity, BID: twice a day, CD: calcium dobesilate, CDDP: compound Danshen dripping pill, includes Danshen (*Salvia. miltiorrhizae*), notoginseng (*Panax notoginseng*), and borneol, CIMT: carotid intima-media thickness, CME: cystoid macular edema, CMT: central macular thickness, CNV-PED complex: choroidal neovascularization-pigment epithelial detachment complex, CSME: clinically-significant macular edema, CSMT: central subfield mean thickness, Diaberet®: antioxidant supplement, each tablet contained 50 mg of pycnogenol® (*Pinus pinaster*), 30 mg of vitamin E and 20 mg of CoQ, DiVFuSS : consists of vitamins C, D3 and E (d-α tocopherol), zinc oxide, eicosapentaenoic acid, docosahexaenoic acid, α-lipoic acid (racemic mixture), coenzyme Q10, mixed tocotrienols/tocopherols, zeaxanthin, lutein, benfotiamine, N-acetyl cysteine, grape seed extract, resveratrol, turmeric root extract* Curcuma longa*, green tea leaf (*Camellia sinensis*) , and Pycnogenol (patented French Maritime Pine Bark extract, sp Pinus pinaster, Horphag Research, Geneva, Switzerland), DPNSS: diabetic peripheral neuropathy symptom scores, DR: diabetic retinopathy, ETDRS: early treatment diabetic retinopathy, FA: fluorescein angiography, fERG: focal electroretinogram, FFA: fundus fluorescein angiography, Fufang Xueshuantong (FXST) formula is composed of *Panax notoginseng* (San-Qi), *Salvia miltiorrhiza* (Danshen), *Astragali radix* (Huang-Qi) and *Radix scrophulariae* (Xuan-Shen), , GSPE: grape seed proanthocyanidins extract, Hachimi-jio-gan (HJG): a chinese herbal formula: 1-*Rehmannia* root 2-*Cornus* fruits 3-*Dioscorea rhizome* 4-A*lisma rhizome* 5-*Hoelen* 6-*Moutan bark* 7-*Cinnamon bark *8-*Aconite*, HBOD: Huangban Bianxing One decoction, comprised eight herbs including: *Astragalus propinquus*, *Angelica sinensis*, *Panax notoginseng*, *Typha angustifolia pollen*, *Fritillaria thunbergii*, *Radix curcumae wenyujin*, *Wolfiporia extensa, Radix et rhizome rhei palmati*, HEs: hard exudates, HESA-A: a drug of herbal-marine origin,1-*Carum carvi* (Persian cumin), 2-*Penaeus latisculatus* (king prawn), and 3- *Apium graveolens (*celery*), *Huo Xue Ming Mu decoction: 1-*Radix bupleuri* 2-*fructus gardenia* 3-*cortex moutan radices* 4-*fructus ligustri lucidi* 5-*Semen plantaginis* 6- *Flos eriocauli* 7-*Radix Salvia miltiorrhiza* 8-*Rhizoma chuanxiong* 9-*Flos chrysanthemi* 10-*Ophicalcitum *11-*Fructus leonuri* 12-*Radix notoginseng*, IOP: intraocular pressure, LDP and GLT: Liuwei Dihuang Pill*, Ginkgo* leaf tablet. Liuwei Dihuang: 1-*Rehmanniae radix praeparata*, 2-*Corni fructus*, 3-*Dioscoreae rhizoma*, 4- *Alismatis rhizoma,* 5-*Moutan cortex*, and 6-*Poria* (the proportion was 8: 4: 4: 3: 3: 3)* Ginkgo* leaf tablet: *Ginkgo biobla*, Meriva®: An herbal medication composed of one-part curcuminoids, two parts lecithin from nongenetically modified soy, and two parts of microcrystalline cellulose. Each tablet containing 500 mg *Meriva®* corresponding to 100 mg *curcumin*, MN: Mean defect, MPOD: macular pigment optical density, NAION: Non-arteritic anterior ischemic optic neuropathy, NPDR: non-proliferative diabetic retinopathy, NVAMD: neovascular age-related macular degeneration, Pycnogenol®: a natural plant extract originating from the bark of the maritime pine., Qiming granule: *Radix astragali, Radix puerariae, Radix rehmanniae,* and *Fructus Lycii* etc, RNFL: retinal nerve fiber layer, ROS: reactive oxygen species, RVO: retinal vein occlusion, S.C Injection: subcutaneous injection, TID: three times a day, TMV: total macular volume, VAR: venoarteriolar response, VEP_S_: visual evoked potentials, VF: visual field, VGEF: vascular endothelial growth factor, Zeaxanthin: The pigment that gives paprika (made from1 -bell peppers), 2-corn*,* 3-*saffron,* 4-wolfberries, and many other plants and microbes their characteristic color, ZQMT*: *Zhixue Quyu Mingmu tablet, composed of 12 herbs including *Rheum officinal, Panax notoginseng, Salvia miltiorrhiza, Eclipta prostrata, Ilex pubescens, Rehmannia glutinosa, Paeoniae rubra, Paeonia suffruticosa, Scutellaria baicalensis, fructus ligustri lucidi, *and* Semen leonu*.

**AMD**

11 studies were dedicated to AMD patients, 3 studies were evaluated herbal medicines as adjunctive therapy with Ranibizumab (0.5 mg per month, intravitreal injection), and all three studies were significantly effective in improving visual acuity (Jin et al., 2018; Luo et al., 2019; Pan et al., 2020). However, Zhixue Quyu Mingmu Tablet (ZQMT), which consists of 12 herbal medicines, did not show a significant difference in visual acuity compared to the Ranibizumab group. But, it was reported very useful for the neovascular AMD patient in significant reduction of hemorrhage, fluid, and lesion size, as well as in reducing the times required for Ranibizumab injection ((p<0.0001) and reducing the cost (Jin et al., 2018). The Huangban Bianxing One decoction (HBOD) comprised of eight herbs also showed beneficial effects in reducing bleeding, CMT and lesion, and also showed a more significant improvement ( p<0.01) in visual acuity compared to the control group; however, the control group received only Ranibizumab without placebo (Luo et al., 2019). In a randomized double-blind study, Fufang Xueshuantong capsules containing *Panax notoginseng*, *Salvia miltiorrhiza* (Danshen), *Astragali radix*, and *Radix scrophularia* showed a significant reduction of choroidal neovascularization-pigment epithelial detachment complex (CNV-PED) compared to the Ranibizumab group in AMD patients (p<0.05)(Pan et al., 2020).

fERG measure was one of the main variables studied in three clinical studies evaluating the effectiveness of saffron (*Crocus sativus*) in AMD patients. Administering 20 mg of saffron tablets showed significant results in improving vision. After 15 months of follow-up in the longitudinal study, an open-label intervention showed improvement in mean fERG sensitivity and visual acuity after three months )p<0.01)(Piccardi et al., 2012). In a placebo-controlled randomized study, evidence showed that the amplitude and functions of fERG increase over 12 weeks in the saffron treatment group compared with placebo (Falsini et al., 2010). Although fERG improved in AMD patients compared to baseline after treatment with saffron, no significant differences were reported in the CFH (Complement Factor H) or ARMS2 (Age-Related Maculopathy Susceptibility 2) polymorphism genotypes. CFH and ARMS are genes that affect the progression of AMD disease (Marangoni et al., 2013).

A clinical trial of daily administration of 13.7 gr of milk-based *Lycium barbarum* L showed that antioxidant capacity was positively correlated with zeaxanthin levels, resulting in a significant increase in the rate of drusen and hypopigmentation compared to the placebo group (Bucheli et al., 2011). Oral daily administration of 15 grams of *Lycium barbarum* L, which is equivalent to 3 grams of zeaxanthin and lutein during 28 days, increased the plasma concentration of zeaxanthin compared to placebo in a single-blinded study (Cheng et al., 2005). 3R, 3R -zeaxanthin dipalmitate vs. non-esterified 3R, 3R՜-zeaxanthin based on milk formulation showed higher bioavailability (Breithaupt et al., 2004). Formula ((A)) oral powder sachet containing *Lycium barbarum* L, which was homogenized in skimmed milk at 80°C, also showed higher bioavailability compared to other formulations B, containing wolfberries homogenized in skimmed milk at 40°C and formulations C, which contained wolfberries that had been ground and heated in water at 80°C (Benzie et al., 2006). HESA-A, a formulation with marine plant origin, also showed beneficial results in improving the visual acuity during the 5-month study compared to placebo (Ahmadi et al., 2009).

**Diabetic retinopathy**

Majority of included articles (14 studies) reported the efficiency of herbal therapy in patients with DR, of which 7 were specific to NPDR patients, and in one study, in addition to DR, other diseases of retinal vascular disorders were examined.

Crocin, the main active ingredient of saffron, was used in patients with refractory diabetic maculopathy. Conventional treatments including macular photocoagulation and intravitreal injection of anti-VEGF agent (bevacizumab) with or without steroids (triamcinolone) were studied in 3 groups. Findings showed that daily administration of 15 mg of crocin compared to placebo and crocin 5 mg reduced CMT (p=0.005) and improved visual acuity (p=0.012) (Sepahi et al., 2018). Retinal sensitivity in macular cystoid edema (CME) patients without macular thickness was reduced during a randomized clinical study with the treatment group receiving an oral combination of desmin (300 mg/day) and troxerutin (300 mg/day) with *Centella asiatica* (30 mg/day) and Melilotus (160 mg/day) for 14 months compared with the control group (p<0.001). However, it had no significant effect on macular thickness and visual acuity (Forte et al., 2011).

Meriva®, a lecithinized curcumin delivery system that contains 100 mg of curcumin per pill, showed a significant improvement in retinal edema during a 4-week randomized study compared with controls in DR. Also, during a 12-week study, in addition to improvement in edema and visual acuity, the thickness of the central macula was reduced compared to the baseline in diabetic macular edema (DME) patient (p<0.01) (Mazzolani et al., 2018; Steigerwalt et al., 2012). Moreover, Liuwei Dihuang Pills (LDP) (*Rehmanniae radix* praeparata, *Fructus Corni*, *Dioscoreae rhizoma*, dried rhizome of *Alisma orientale*, and root bark of *Paeonia suffruticosa* Andr) and Ginkgo Leaf Tablets (GLT) resulted in a significant improvement of vascular complications, diabetic neuropathy, and DR prevalence (p<0.050) in patients with type 2 diabetes. Despite their beneficial effects, further investigation is necessary due to the risk of macrovascular complications (Zhao et al., 2016). The DiVFuSS consisted of some vitamins and minerals and combination of this medication with herbal products such as Pycnogenol (*Pinus pinaster*), zeaxanthin, lutein, and grape seed extract, resveratrol turmeric root extract, and green tea leaf could provide significant improvements in visual function. Macular pigment optical density (MPOD), one of the main variables in a double-blind clinical trial, was increased in the treatment group after treatment with DiVFuSS capsule (Chous et al., 2016). Studies have shown that administration of Pycnogenol® French maritime pine bark extract (*Pinus pinaster*), three times a day, reduce retinal edema and improve visual acuity in patients with DR, and changed the vascular pattern and endothelial permeability of the treatment group compared to baseline. Pycnogenol® could also affect hemorrhages, hard exudates (HEs) in the macula center, and visual acuity (Steigerwalt et al., 2009). 

Clinical assessment of retinal blood circulation in patients with DR treated with Qiming granule consisting of extracts from several medicinal plants including *Radix Astragali*, *Radix Puerariae*, *Radix Rehmanniae*, and *Fructus Lyci* demonstrated a significant reduction in the retinal arterio-venous circulation time (AVCT) in the treatment group (p<0.01) compared to the control group receiving calcium dobesilate capsule, suggesting that this herbal medication can improve retinal hypoxia and ischemia (Luo et al., 2009).

Recent evidences show that treatment of NPDR patients with *Abelmoschus manihot*, Jiahua tablet (1.8 gr per day) improved the remission rate of DR and the Early Treatment Diabetic Retinopathy (ETDRS) vision score. Also, the cube average thickness (CAT), central subfield thickness (CST), cube volume (CV) (p<0.0001), and serum VEGF levels were significantly improved (p<0.0026), and the progression rate of DR in the treatment group were reduced after six months of treatment. It also improved the ETDRS vision score (p=0.0002) and significantly reduced the rate of progression of NPDR severity (p=0.007) in the treatment group (Zhao et al., 2020). Safety assessment of orally administered grape seed proanthocyanidin extract (*Vitis vinifera*) (GSPE) as a source of proanthocyanidin similar to Pycnogenol® (*Pinus pinaster*) was evaluated in 124 patients for 12 months of follow-up, and the results demonstrated that administration of 150 mg/day of GSPE has higher efficacy for management of HEs compared to calcium dobesilte (CD; 750 mg/day). The rate of improvement was significantly higher in the GSPE group than in the CD group (Moon et al., 2019). Danshen dripping pill (CDDP) comprises the extract from Danshen (*Salvia miltiorrhiza*), and notoginseng (*Panax notoginseng*), and each CDDP pill contains 27 mg of a mixture of herbal compounds, which has an evident therapeutic effect to calcium dobesilate on early DR, suggesting that CDDP can be used for the management of microaneurysm and hemorrhage to improve visual acuity and visual field (p<0.05). At the high- dose (30 tablet a day equivalent to 810 mg/day) and mid-dose (20 tablet a day equivalent to 540 mg/day), fundoscopic examination demonstrated significant improvement in CDDP groups compared to the placebo group (p<0.001) at 24 weeks. The BCVA, mean defect of the visual field, hemorrhage area of the fundus, microaneurysm number, fluorescent leakage area, and capillary nonperfusion area were significantly improved in the treatment group, indicating that CDDP has similar efficacy and safety to calcium dobesilate for NPDR, and suggested that CDDP may function as the auxiliary drug (Lian et al., 2015; Luo et al., 2015). Circulating levels of reactive oxygen species (ROS) and changes in CMT after administration of antioxidant supplementation (Diaberet® tablet) containing pycnogenol® (50 mg), vitamin E (30 mg), and coenzyme Q10 (20 mg) were significantly reduced (p<0.001) (Domanico et al., 2015).

**Retinitis pigmentosa**

The results of 12-month intervention on 42 patients with retinitis pigmentosa (RP) demonstrated that *Lycium barbarum *L. (LB) oral supplement (two packs/day) can lead to contrast visual acuity in the LB group compared to the placebo group (p=0.001). Thinning of the macular layer was also observed in the placebo group, but not in the treatment group (p=0.008). However, there was no significant difference in visual field sensitivity or any fERG parameters between the two groups. In addition, no significant side effects were reported in the treatment group (Chan et al., 2019).

**Retinal vasculature disorder**

Administration of Hachimi-jio-gan (HJG), a Chinese herbal formula (BID), significantly increased the systolic and diastolic flow velocity and the mean flow velocity in the central retinal artery (p<0.05) (Isobe et al., 2003). Pycnogenol (*Pinus pinaster*) was another important and effective medicinal plant that demonstrated significant recovery of visual acuity, improvement of retinal vascularization and reduction of endothelial permeability (Domanico et al., 2015).

**Stargardt disease**

A randomized, double-blind, placebo-controlled clinical study showed that daily administration of 20 mg of saffron over 20 weeks prevents fERG amplitude changes in the saffron group, whereas fERG amplitude decreases in the control group. Although saffron was not effective in improvement of fERG, it could reduce the progression of the Stargardt disease (STG) (Piccardi et al., 2019).

**Non-arteritic anterior ischemic optic neuropathy**

Safety assessment of RPh201 (20 mg) (a botanical extract of gum *Pistacia lentiscus*), administered subcutaneously twice weekly for 26 weeks for improving visual function in patients with previous non-arteritic anterior ischemic optic neuropathy (NAION) did not raise any safety concerns (Rath et al., 2019).

## Discussion

The eye is an organ with a high metabolic rate, which suffers from consistent photo-oxidation by the light. Ocular tissue cells are sensitive to oxidative damage, and there are many pathological factors associated with eye diseases, such as age, genetic susceptibility, oxidative stress, inflammation, and tumorigenesis. Angiogenesis, ischemia, diabetes, and oxidative stress seem to play a key role in the pathogenesis of eye diseases (Xu et al., 2018). The main goals of treatment are to prevent or slow disease progression and preserve, improve or restore the vision. In several cases, the harm that has already occurred cannot be reversed. Conventional treatments of the retinal disease include laser therapy, cryopexy, injecting air or gas into the eye, and injecting drugs into the eyes, which are effective in treating individuals with wet macular degeneration, DR, or broken blood vessels in the eye (Piccardi et al., 2019). Many herbal remedies have been introduced for treatment of eye diseases, as recorded in numerous ancient and modern Chinese pharmacopoeias. The effectiveness of those remedies relies on experiences and separate clinical outcomes, without strictly passing through modern systematic scientific assessments together with animal testing and clinical trials. Furthermore, the efficacies have additionally been supposed as having an outsized variation due to no standardized sources, qualities, combinations, and preparation strategies of herbal remedies. Nevertheless, these long-documented historical remedies are a goldmine for the natural product (Xu et al., 2018). Medicinal herbs as alternative and adjuvant therapies were effective in reducing retinal cell apoptosis, improving retinal endothelial cell function, and inhibiting neovascularization (Lian et al., 2015). The results of this review showed that herbal therapy might be beneficial for the treatment, prevention, and adjutant therapy on AMD, DR, or broken blood vessels at the retina in the eye. Most of the patients under treatment with herbal medications showed significant improvement on CVA, fundus performance, retinal thicknesses, fundoscopic examination parameters such as hemorrhage, and other variables compared to the patients in the placebo group in therapy duration of 3 to 144 weeks. Besides, efficacy was observed in the non-retinopathy variable. These herbal medicines have been used in various clinical studies alone or combined with other medications.

**AMD**

AMD is a neurodegenerative disease characterized by soft drusen and hyper-hypopigmentation of the retinal pigment epithelium in the early stages of the disease, and by retinal pigment epithelium atrophy or subretinal neovascular membranes in the later stages (Mitchell et al., 2018). Effective medicinal plant for the treatment of AMD was from the most studied medicinal plants *Lycium barbarum* (especially zeaxanthin, a potential antioxidant) that increased fasting plasma zeaxanthin and antioxidant levels and protected the macula from hypopigmentation and soft drusen accumulation (Bucheli et al., 2011; Cheng et al., 2005). Also, lutein besides the zeaxanthin was shown to reduce the risk of AMD. In terms of bioavailability, the formulation containing zeaxanthin, which was homogenized in skimmed milk at 80°C, showed higher bioavailability (Benzie et al., 2006). Another most studied medicinal plant was Saffron and its active ingredient (crocin), which were prescribed as a supplement (saffron 20 mg/day or crocin 15 mg/day) in patients with AMD and could significantly improve fERG amplitude, fERG sensitivity, and visual acuity (Falsini et al., 2010). Saffron seems to have a neuroprotective effect on retinal cells and also prevents the effect of TNF-α- induced apoptosis by modulating Bcl-2 protein expression and suppressing caspase activity (Fernández-Albarral et al., 2020). Treatment of AMD patients with HESA-A (a drug of marine plant origin) tablet could also improve visual acuity compared to the baseline, and showed superior willingness due to its efficacy, simple oral usage, and short treatment period compared to long-term VEGF drugs therapy. Studies have shown that these effects may be mediated by their anti-inflammatory and antioxidant effects; however, the clinical trial measured only visual acuity (Ahmadi et al., 2009). 

Anti-VEGF agents such as ranibizumab are approved by the US Food and Drug Administration (FDA) and European Medicines Agency for treatment of DME, DR, Retinal Vein Occlusion (RVO), and AMD. Endothelial growth factor-inhibiting aptamers are small, stable RNA molecules that bind to human VEGF and inhibit its excessive release, which prevents deep arteries to become permeable and fluid to leak from new arteries. Therefore, anti-VEGF agents prevent retinal damage, severe edema and vision loss (Petri et al., 2020). However, several possible side effects have been reported such as a transient increase in intraocular pressure (IOP) and endophthalmitis after intravitreal injection (Forte et al., 2011; Katsi et al., 2012). Also, some patients may experience eye irritation and pain after 24 hours of intraocular injection. There is also initial clinical evidence that there is a possibility of increased stroke in AMD patients under treatment with the bevacizumab (Katsi et al., 2012). Combination of ranibizumab (0.05 ml; 10 mg/ml per dose) with ZQMT, which consists of 12 herbal medicines (administration with 12 tablets per day) or HBOD decoction administered as oral solution (100 ml BD) or daily oral supplementation of cFXST (*Panax notoginseng*, *Astragalus membranaceus*, *Salvia miltiorrhiza*, and *Scrophularia ningpoensis*) as adjunctive therapy showed improvement in visual acuity. In between, HBOD decoction and ZQMT tablets were effective in reducing visual acuity by reducing bleeding, and cFXST supplementation was effective in reducing visual acuity by reducing choroidal neovascularization. *Panax notoginseng* was a common plant in all three formulations of this drug and was effective in reducing and absorbing bleeding. Also, *Salvia miltiorrhiza* was used in both HBOD and cFXST formulations, which may play an anti-inflammatory role in improving blood flow (Luo et al., 2019; Pan et al., 2020). The major therapeutic advantages of ZQMT were the reduction of retinal lesions and the financial burden of patients undergoing treatment by reducing the frequency of ranibizumab injections (Jin et al., 2018).

**Diabetic retinopathy**

Early-onset DR is usually asymptomatic and is diagnosed by functional changes in electroretinography, retinal blood flow, and retinal blood vessel caliber, as well as clinical factors in the retinal fundus by ocular fluid. DR is divided into proliferative and non-proliferative retinopathy based on the absence of blood vessel lesions and neovascularization (Lechner et al., 2017). CME is a complication of DR, measured by Optical coherence tomography (OCT) in normal thickness and focal contour (Jun et al., 2010).

Combination of flavonoids desmin and troxerutin with *C. asiatica* and Melilotus has been shown to decrease endothelial permeability and capillary filtration, which is active in diabetic microangiopathy. It also reduces fascial edema in these patients. Although changes in BCVA and central retinal thickness (CRT) were significant compared to controls, significant retinal sensitivity changes were reported after 14 months follow-up in CME patients without macular thickening (Forte et al., 2011). Another study on microangiopathy and retinopathy demonstrated that the administration of Meriva® formulation, which is a solid form of lecithin dispersed curcumin, has higher adsorption than curcumin alone, indicating that the herbal medication composed of curcuminoids, lecithin, and microcrystalline cellulose significantly improved peripheral microangiopathy and reduced the score of peripheral edema, leading to improvement in visual acuity in chronic DME (Steigerwalt et al., 2012). Curcumin targets inflammation and edema with pleiotropic anti-inflammatory activity as well as by inhibiting the local effect of VEGF factors. This mechanism along with lifestyle changes help to treat the diabetes and diabetes-induced eye diseases in diabetics, pre-diabetics, and patients with chronic macular edema (Isobe et al., 2003). Crocin, the active ingredient in saffron, is known as an antioxidant supplement, which can reduce the sensitivity of retinal cells to the dangers of inflammation induced by oxidative stress. Studies have shown a beneficial effect of crocin in reducing inflammation and thus reducing macular thickness in patients with DME. Crocin prevents ischemia by inhibiting apoptosis through the PI3K/AKT signaling pathway in retinal ganglion cells. Clinical data showed that Liuwei Dihuang Pills (LDP) and Ginkgo Leaf Tablets (GLT) are beneficial in the management of diabetic microvascular complications (Zhao et al., 2016). The DiVFuSS consisted of some vitamins and minerals, and a combination of this medication with herbal products such as Pycnogenol (*Pinus pinaster*), zeaxanthin, lutein, grape seed extract (*Vitis vinifera*), resveratrol, turmeric root extract (*Curcuma longa*), and green tea leaf (*Camellia sinensis*) could provide significant improvements in visual function by improving macular pigment optical density. Anti-apoptotic properties and antioxidant effects of some herbal products are the major underlying mechanism involved in therapeutic effects of medicinal plants in the treatment of eye diseases. Also, anti-VEGF properties of curcumin, green tea, and zeaxanthin/lutein have been reported as the main mechanism behind therapeutic effects in animal models of eye diseases (Chous et al., 2016).

The composition of Pycnogenol®, which is prepared from pine bark extract (*Pinus pinaster*), improves visual acuity in patients with DR, especially in the early stages of the disease through anti-inflammatory, antioxidant, and protective properties of retinal arteries by binding to proteins and capillary wall to reduce capillary permeability (Spadea and Balestrazzi, 2001; Steigerwalt et al., 2009). Also, Diaberet® supplement, containing 50 mg of pycnogenol® (*Pinus pinaster*), 30 mg of vitamin E, and 20 mg of coenzyme Q10, is an antioxidant supplement that can reducethe macular thickness and oxygen levels of free radicals (Domanico et al., 2015). The exact mechanism of improving visual function by grape seed extract Proanthocyanidins (GSPE) (*Vitis vinifera*) is not known, but it seems that metabolic management has the major role for beneficial effects of these products in reducing hard exudates compared to calcium dobesilate and controls (Moon et al., 2019).

Calcium dobesilate -calcium salt of dobesilic acid- is used for the treatment of DR. Reducing retinal albumin leakage and capillary permeability, which protects the blood-retinal barrier (BRB), inhibiting platelet aggregation and blood viscosity, and increasing endothelial-dependent relaxation are the major mechanisms behind the therapeutic effects. It also upregulates nitric oxide synthase, inhibits apoptosis of vascular endothelial cells in blood vessels, and protects against ROS through its antioxidant and antiradical activity. In addition, calcium dobesilate inhibits the expression of the upstream and inflammatory VEGF regulator ICAM1 (Liu et al., 2019; Rota et al., 2004). Qiming granule contains *Radix astragali*, *Radix puerariae*, *Radix rehmanniae*, *Fructus lycii*, etc., which could alleviate retinal hypoxia and ischemia by increasing retinal blood flow and improving the blood circulation in diabetic patients; Qiming granules in a study with a control group that consumed calcium dobesilate showed that changes in AVCT were more significant in the treatment group. Its possible mechanism is through improving the circulation of the retinal microvascular system, which includes improving blood flow, reducing hypoxia and ischemia, and protecting the retinal blood vessel. Animal studies have shown that *Radix astragali*, *Radix rehmanniae*, and *Radix puerariae* protect retinal blood vessels by protecting blood cells against abnormalities, reducing clotting and fibrin, and improve blood flow by reducing cell aggregation (Luo et al., 2009). The therapeutic value and safety of danshen-containing Chinese herbal medicine (*Salvia miltiorrhiza*) were also demonstrated in these patients. Findings of a study showed that CDDP has an evident therapeutic effect on calcium dobesilate on early DR, suggesting that CDDP can be used for the management of microaneurysm and hemorrhage to improve visual acuity and visual field. At high doses of 810 mg and medium doses of 540 mg, it had an excellent and very good performance, respectively (Lian et al., 2015; Luo et al., 2019).

It has been suggested that possible mechanism of therapeutic action for *Abelmoschus manihot* as a supplement may be through improving blood flow and reducing apoptosis and protecting the function of retinal endothelial cells, which can reduce the progression of retinopathy and improve visual acuity in patients with NPDR (Zhao et al., 2020).

**Retinitis pigmentosa**

RP is a hereditary disease that is caused by loss of light receptors, and the diagnosis is based on clinical signs such as night blindness, peripheral visual field defects, fundus lesions, and abnormal electroretinogram (Hamel, 2006). One of the treatment strategies for this disease is the preservation of photopic vision. *Lycium barbarum* polysaccharides are components with antioxidant activity that have been shown to protect optical receptors against degeneration, apoptosis, and oxidative inflammation in animal models (Chan et al., 2019).

**Retinal vasculature disorder**

RVO can be caused by diabetes, hypertension, atherosclerosis, in some cases thromboembolism, or anatomical abnormalities. Depending on the extent and type of obstruction, patients may lose their sight for a variety of reasons such as interrupted blood flow to the macula, ischemia, optic neuropathy, bleeding, and retinal detachment. HJG tablet (27 mg twice a day) is a Chinese herbal formula consisting of 1-Rehmannia Root *(Rehmannia glutinosa) 2-*Cronus Fruits 3-Dioscorea Rhizome 4-Alisma Rhizome* (Alisma orientale*) 5-Moutan Bark (*Paeonia suffruticosa Andr*) 6-Cinnamon Bark (*Cinnamomum cassia*) 7-Aconite (*Aconitum*) that has been shown to increase blood flow in the central retinal artery, indicating the desired therapeutic effects of HJG on eye diseases such as DR (Isobe et al., 2003). Similarly, Huo Xue Ming Mu decoction consisting of 1-*Radix bupleuri* 2-*Fructus gardenia* 3-*Cortex moutan radices* 4-*Fructus ligustri lucidi* 5-*Semen plantaginis* 6- *Flos eriocauli* 7-*Radix Salvia miltiorrhiza* 8-*Rhizoma chuanxiong* 9-*Flos chrysanthemi* 10-*Ophicalcitum* 11-*Fructus leonuri* 12-*Radix notoginseng* demonstrated remarkable effects on retinal vein obstruction. It is more effective in reducing bleeding than CDDP that contains *S. miltiorrhizae* and notoginseng (*Panax notoginseng*) (Xu et al., 2018; Zhu et al., 2002).

**Stargardt disease**

Stargardt disease is one of the most common genetic diseases with macular dystrophy, the symptoms of which are characterized by central vision disorder (bilateral macular atrophy) appears in adolescence and youth (Tsang and Sharma, 2018). Saffron has been shown to be effective in the management of this disease. Findings suggested that although the amplitude of fERG was not changed in the saffron treatment group, a significant progression of the disease process was observed in the control group. This data showed that saffron was effective in reducing the progression of the disease (Piccardi et al., 2019).

**Non-arteritic anterior ischemic optic neuropathy**

NAION, which results in unilateral loss of blood supply to the optic nerve leading to unilateral vision loss, is generally painless (Berry et al., 2017), and its pathophysiology is not known. In these patients, RPh201 (a botanical extract of gum *Pistacia lentiscus*) could improve BCVA after 26 weeks of treatment. The most frequently reported adverse effect was injection site pain (Rath et al., 2019).

In summary, out of 30 selected articles, 11 articles evaluated the therapeutic effects of herbal products in the treatment of AMD, and 3 studies evaluated herbal medication as adjuvant therapies with the Ranibizumab. Major therapeutic mechanisms were the preservation of macula and pigment density through antioxidant effects, control and management of inflammatory factors, and maintaining the thickness of the central macula to prevent edema and hard exudates. Also, visual acuity and fERG amplitude were the other variables that were measured in these studies. The concentration of zeaxanthin as a natural pigment was another variable studied for patients receiving *Lycium barbarum* L (Cheng et al., 2005). The other 14 articles evaluated patients with DR, macular edema, and NPDR. The overall treatment mechanism was controlling blood flow to prevent abnormal angiogenesis and ischemia. Three studies compared the efficacy of herbal supplements with calcium dobesilate in the management of retinal vascular blood supply and protection of retinal blood vessels. Changes in macular thickness and visual acuity were also among the other main variables measured. The other five studies evaluated the effectiveness of herbal therapy for other retinal vascular disorders, RP, and NAION.

In general, combination formulations due to acting through various mechanisms of each plant had more beneficial results as a supplement. Saffron showed beneficial effects mainly in AMD patients, DR, and Stargardt disease. Supplements and adjuvant medications appear to be more beneficial in patients with AMD and diabetic maculopathy. Although studies have shown the usefulness of herbal remedies in the early stages of DR and NPDR, they may also be helpful in preventing the progression of degeneration in addition to reducing the thickness of lesion and subsequent edema. The important limitation of this study was the limited number of clinical trials, most of which were not in the English language; therefore, we avoided article exclusion as much as possible to minimize data loss. Also, the severity of the disease and treatment protocols, and the effectiveness of each plant in the combination of the herbal product had not been reported in some studies, so the results were only described in general, which may increase the risk of bias. 

The results of this systematic review indicated that medicinal plants and herbal products alone or in combination with other medications can be considered as a potential therapeutic approach for the treatment of eye diseases, especially in the first stages of retinopathy. Higher efficiency, lower side effects, and more acceptability by patients were the main advantages of herbal medications. However, more clinical evidence is needed to verify such efficacy, and safety.

## Conflicts of interest

The authors have declared that there is no conflict of interest.
